# Evaluation of Knowledge, Attitude, and Practice (KAP) of Artificial Intelligence Among Dentists and Dental Students: A Cross-Sectional Online Survey

**DOI:** 10.7759/cureus.44656

**Published:** 2023-09-04

**Authors:** Gnanambigai Kalaimani, Sivapathasundharam B, Rajeswari M Chockalingam, Prem Karthick

**Affiliations:** 1 Oral Pathology, Priyadarshini Dental College and Hospital, Chennai, IND; 2 Oral Pathology and Microbiology, Priyadarshini Dental College and Hospital, Chennai, IND; 3 Oral and Maxillofacial Pathology, Priyadarshini Dental College and Hospital, Chennai, IND

**Keywords:** dental students, dental surgeons, dental curriculum, research, deep learning, artificial intelligence

## Abstract

Background: Artificial intelligence (AI) is the process by which it is possible to program computers to mimic human thoughts. AI and its subsets machine learning and deep learning have been developed to analyze complicated data gathered from many sources using algorithms built into decision support systems. It has been widely used in the field of dentistry.

Aim: The study aimed to evaluate the knowledge, attitude, and practice (KAP) of AI among dental students and dentists.

Methodology: The present study is a descriptive cross-sectional online survey that was carried out among dentists and dental students in South India. A self-structured, close-ended questionnaire that was administered that consisted of 25 questions was included. The questions were circulated through Google Forms (Google LLC, Mountain View, California, United States), and it was circulated among the study participants through online mode. The data were collected systematically, and SPSS Statistics version 22.0 (IBM Corp. Released 2013. IBM SPSS Statistics for Windows, Version 22.0. Armonk, NY: IBM Corp.) was used for data analysis.

Results: One thousand (595 dental surgeons and 405 dental students) participated in the study through Google Forms. Among these, 700 (70%) were females and 300 (30%) were males. In the study group, 635 (63.5%) were aware of AI, and 365 (36.5%) were not aware (p-value 0.000). Among the 21 questions used to assess the KAP, 14 questions were significant with a p-value less than 0.05. More than 60% agreed that the dental curriculum has to be updated with AI. About 269 (26.9%) agreed that AI will replace the role of dentists in the future. There were no significant results in comparing dental surgeons and dental students.

Conclusion: The present study showed that the KAP among dental surgeons and dental students was the same. They believe that the dental curriculum has to be updated with AI. This study shows that there is a lack of knowledge about deep learning models and websites used for AI among dentists. Thus, it is necessary to include evidence-based teaching and training about the application of AI in dental practice to improve the future of dentistry.

## Introduction

John McCarthy in 1956 first proposed the applied computer science known as artificial intelligence (AI). It is the reproduction of human intelligence in devices that have been designed to reason and acquire knowledge like humans. Robotics, computer vision, machine learning, and natural language processing are all sub-fields in this branch [1]. AI is used in various applications in the healthcare industry, including decision-making, improved business processes, higher quality, monitoring and delivering individualized treatment regimens, and many more [2]. AI has the potential to advance both the medical and dental disciplines. Applications of AI in the dental industry are not yet prevalent, but the development of these technologies had an impact on robotic assistance, dental image diagnostics, caries detection, radiography and pathology, and electronic record keeping [1].

A core curriculum of dental education needs to be updated in this fast-changing environment since AI in healthcare is fundamentally changing the methods utilized in diagnosis, treatment plans, and prognosis [3]. The various subsets of AI include machine learning which is used to predict results out of a data set, making it easier for machines to acquire data already available and solve problems without any human intervention. Similar to that of the human brain neural networks work by the use of artificial neurons and compute signals [4]. In deep learning, numerous computational layers create a network of neurons that identifies patterns on their own and detects them [4].

In research writing, AI tools have been broadly classified into two categories. One is that supports writers throughout the writing process and the other one is those that review and assess the quality and validity of written material [5]. AI improves both efficiency and time management in research writing. Natural language processing algorithms enable authors to focus on the content of their writing rather than correcting their errors. These algorithms also give specific outlines for manuscripts, research protocols, informed consents, grant proposals, emails, and other documents [5]. The present study aimed to assess the knowledge, attitude, and practice (KAP) of AI among dental students and dental surgeons.

## Materials and methods

Methodology

To assess the KAP among dental students and dental surgeons in various districts of Tamil Nadu, a cross-sectional questionnaire survey was conducted through Google Forms (Google LLC, Mountain View, California, United States). The Institutional Ethical Committee of Priyadarshini Dental College and Hospital issued approval IEC-PDCH 4/2 2023. After obtaining ethical clearance, a self-structured questionnaire consisting of 25 questions was framed. Among these first four questions were about demographic details including age, gender, and qualifications. The remaining 21 questions were used to assess the knowledge about AI. One thousand randomly selected dental surgeons from registered dental councils and dental students from various parts of Tamil Nadu participated in this survey through Google Forms. About 595 dental surgeons and 405 dental students were included in the study. Among the 595 dental surgeons, 433 were Bachelor of Dental Surgery (BDS), 96 were Master of Dental Surgery (MDS), and 66 were post-graduate students. All the participants were explained the purpose of the study and informed consent was obtained through Google Forms.

Statistical evaluation

Random sampling techniques and cross-sectional design were used to analyze the KAP among 1,000 individuals. Results were evaluated using SPSS Statistics version 22.0 (IBM Corp. Released 2013. IBM SPSS Statistics for Windows, Version 22.0. Armonk, NY: IBM Corp.). Descriptive statistics were used. To compare between dental surgeons and dental students, a chi-square test was used. A p-value less than 0.05 was considered significant.

## Results

A total of 1,000 responses from dental students and dental surgeons were collected. The study group falls within the age group of 18-60 years. About 825 (82.5%) were within the age group of 18-25 years. Among the study groups, 700 (70%) were females and 300 (30%) were males. About 595 dental surgeons and 405 dental students were included in the study. Among the 595 dental surgeons, 433 (43.3%) were BDS, 96 (9.6%) were MDS, and 66 (6.6%) were post-graduate students. Among the 21 questions used to analyze the KAP, 14 questions were significant.

Six hundred thirty-five (63.5%) were aware of AI and the remaining 365 (36.5%) were not aware of AI with a significant p-value of 0.000. Among the websites used for AI, only 372 (37.2%) were aware of the AI apps and the remaining 628 (62.8%) were not aware of what app to use specifically (p-value 0.000). About 534 (53.4%) used AI to specifically improve knowledge about a particular topic, 394 (39.4%) said no, and 72(7.2%) answered don’t know (p-value 0.001). The various large language models (LLM) app usage among dental surgeons and dental students was significant (p-value 0.001). The awareness of the various subsets of AI such as deep learning, recurrent neural networks, convolutional neural networks, and machine learning between the groups was significant (p-value 0.002) (Table 1). Among the students, the use of AI in theoretical answer preparations, practical assignments, and pre-clinical works was significant (p-value 0.000). Among the researchers, the usage of AI in all the stages of research was significant (p-value 0.000). Among the practitioners, the use of AI in diagnosis, treatment plans, prognosis, maintenance of records, follow-ups, and clinical management was significant (p-value 0.000). Seven hundred (70%) agreed that training must be given in AI to all dental students (Table 2).

**Table 1:** Responses to questions AI: artificial intelligence, LLM: large language models

S. NO	Questions	Responses	Total (n%)	p-value
1	Are you aware of AI?	Yes	63.5	
		No	24.8	0.000
		Don’t know	11.7	
2	Do you know the websites used for AI?	Yes	37.2	
		No	45.7	0.000
		Don’t know	17.1	
3	Have you used AI to improve your knowledge regarding a topic?	Yes	53.4	0.001
		No	39.4	
		Don’t know	7.2	
4	What LLM app do you use if yes?	BERT	2.4	
		Galactica/OPT (Meta)	6.3	
		GPT3 (OpenAI)	15.9	0.001
		Megatron (Microsoft)	6.4	
		PaLM/LaMDA (Google)	22.1	
		No	46.9	
5	Are you aware of deep learning models like recurrent neural networks and convolutional neural networks?	Yes	20.2	0.002
		No	54.0	
		Don’t know	25.8	
6	If you are a student, do you know AI can help in theoretic answer preparations, practical assignments, and pre-clinical works?	Yes	43.2	0.000
		No	26.8	
		Don’t know	30.0	
7	As a researcher, do you think applications of AI in all stages of research can improve the quality of it?	Yes	48.3	
		No	19.9	0.000
		Don’t know	31.8	
8	Do you prefer using AI in radiological diagnosis?	Yes	44.1	
		No	22.4	0.08
		Don’t know	33.4	
9	How often do you use AI apps?	When need arises	26.0	
		Frequently	1.2	0.94
		Seldom	50.4	
		To update myself	12.4	
10	Have you used an AI app for getting awareness on practical assignments?	Yes	19.7	
		No	56.5	0.12
		Don’t know	23.8	

**Table 2:** Responses to questions AI: artificial intelligence

S. NO	Questions			Responses		Total (n%)	p-value
11	Are radiologists to be trained adequately in using AI for the interpretation of diagnosis?			Agree		52.4	
				Disagree		11.7	0.2
				Don’t know		35.8	
12	As a practitioner, do you use AI for diagnosis, treatment plan, and prognosis?			Yes		32.3	
				No		33.7	0
				Don’t know		34	
13	As a practitioner, do you use AI for the maintenance of records, follow-up, and clinical management?			Yes		43.1	0.000
				No		26.7	
				Don’t know		30.2	
14	Do you think pathologists can use AI for the interpretation of the color of lesions, photographs, and analysis of histopathology in the diagnosis of cancer?			Yes		50.1	0.10
				No		13.6	
				Don’t know		36.3	
15	Do you think in the field of prosthodontics, AI-assisted procedures can save clinic time, quality of work, and number of visits?			Yes		50.6	0.5
				No		13.5	
				Don’t know		35.9	
16	Do you think the interpretation of cephalometric analysis and treatment plans in the department of orthodontics is also a part of AI?			Ye		49.0	0.08
				No		14.2	
				Don’t know		36.8	
17	Do you think training must be given in AI for all medical/dental students?			Agree		63.9	
				Disagree		10.1	0
				Don’t know		26	
18	Do you think AI can replace the role of dentists/general practitioners?			Agree		26.9	
				Disagree		41.2	0
				Don’t know		31.9	
19	Do you think the medical/dental curriculum can be updated with AI?			Agree		59.7	
				Disagree		10	0
				Don’t know		30.3	
20	Do you think advancing AI in the medical/dental field can affect creativity in the future?			Yes		45.0	
				No		22.2	0.003
				Don’t know		32.8	
21	Do you think the use of AI in the field of medicine/dental field can violate ethical principles?			Yes		40.5	0.002
				No		18.8	
				Don’t know		40.7	

Two hundred sixty-nine (26.9%) agreed that AI in the future will replace the role of dentists. Four hundred twelve (41.2%) disagreed that AI will not replace the role of dentists, and 319 (31.9%) said they don’t know (p-value 0.000). About 597 (59.7%) agreed that the dental curriculum has to be updated with AI, 100 (10%) disagreed, and 303 (30.3%) answered as don’t know (p-value 0.000). Four hundred fifty (45%) accepted that the advancement of AI in the field of dentistry can affect creativity. About 222 (22.2%) said that AI will not affect creativity, and 328 (32.8%) answered don’t know (p-value 0.003). Four hundred five (40.5%) answered that AI in the field of dentistry can violate ethical principles, and 188 (18.8%) answered that it won’t violate ethical principles (p-value 0.002) (Table 2).

In comparison between dental surgeons and dental students, the use of LLM was significant (p-value 0.004). Questions regarding the ethics violations by using AI in dentistry were significant (p-value 0.000) (Table 3).

**Table 3:** Comparison between dental surgeons and dental students LLM: large language models, AI: artificial intelligence

S. NO	Questions	p-value
1	What LLM app do you use?	0.004
2	Do you think the use of AI in the field of medicine/dental field can violate ethical principles?	0.00

## Discussion

AI enables the creation of intelligent machines. AI has begun to have an impact on diagnostic and treatment modalities in the health sector [6]. AI has made great progress in the field of dentistry, especially in diagnosis, treatment planning and prognosis, landmark detection, and risk assessment [7]. The use of machine learning algorithms aids in interpreting the radiographs, and AI-powered apps help us detect mucosal changes and caries [3]. In the present study, participants’ KAP was assessed using 21 closed-ended questionnaires.

Our findings show that 635 (63.5%) were aware of AI and 380 (38%) were aware of AI apps. A study conducted by Shiva Thulasi et al. [6] and Swed et al. [8] showed that more than 70% were aware of AI. In the present study, only 20.2% were aware of deep learning models like convolutional neural networks and recurrent neural networks, in contrast to the study by Shiva Thulasi et al. [6]. Understanding AI's potential and limitations is essential. Dentists must be aware of what AI can and cannot achieve in this field. Making educated decisions about integrating AI technologies into their practice requires keeping up with the most recent advancements and research.

More than 60% were about LLM, similar to studies done by Shiva Thulasi et al. [6]. Among the researchers, awareness of AI was 483 (48.3%). Similar to this study, Sulthan et al. [9] showed that more than 40% of researchers believed that AI may be useful in clinical research and its related research decisions. Personal assistants (Siri, Alexa, Google Assistant, etc.), automated public transportation, aircraft, and video games are just a few examples of how AI is incorporated into our daily lives [10]. Some elements that may greatly impact awareness of AI in dentistry include exposure to relevant information, professional networks, and continuing education. Dentists who regularly follow field news, attend conferences, and take part in online discussion boards may be more aware of the possible uses of AI.

Cancer is not easily detected in its early stages. Thus, a computer-operated system that can distinguish between benign and malignant cells is used in diagnosis [11]. In the present study, more than 50% agreed that AI is used to assess the color of the lesion and analyze the histopathology of cancer. This is similar to studies conducted by Shiva Thulasi et al. [6], Yu et al. [14], Singh et al. [12], and Yuzbasioglu [13]. Numerous AI systems have been created with deep learning algorithms to recognize changed mucosal lesions, perform automated diagnosis of oral lesions, evaluate bone age, and detect and diagnose tooth decay and periodontal disorders using radiography, according to literature studies [6,12-14]. In this study, only a few dentists agreed that AI could replace the role of dentists in the future. This is similar to the study conducted by Yüzbaşolu [13] and Swed et al. [8]. Among the practitioners, 430 (43%) believed that AI helps in clinical management, similar to that of Sur et al. [15]. The use of AI was favored due to its speed, quality, and accuracy in obtaining real-time data for use in healthcare services.

In the present study, 590 (59%) believed that the dental curriculum had to be updated with AI. More than 75% agreed that AI be included in undergraduate and postgraduate dental education in a study conducted by Emir Yüzbaşıoğlu [13]. The wider healthcare field, including dentistry, has been exploring AI's potential to improve diagnosis, treatment plans, patient management, and administrative tasks. The awareness of AI in future dental practices has been influenced by this tendency. The dentistry curriculum needs to be revised to include AI.

In this study, there is not much difference between dental surgeons and dental students regarding the KAP of AI. This is similar to the study conducted by Shiva Thulasi et al. [6]. Some dentists might see AI as a tool that could improve their patient care, diagnose and treat patients more effectively, and expedite administrative work. Others, however, may be concerned about job loss, the loss of the personal touch in patient care, and potential ethical concerns with the use of AI in dentistry.

Still, the use of AI is not integrated into dentistry. The biggest limitations were inadequate information and a lack of awareness regarding integrating AI. Additionally, the participants were professionals with clinical expertise as well as dental students who may have contributed distinct AI conceptualizations to the overall study output [16,17]. This was in contrast to studies conducted in Turkey by Yüzbaşolu [13] and Korea by Oh et al. [18]. These differences may be the result of varying educational programs and instructional methods in different countries.

The limitation of this study includes the years of experience among students and dentists, which have varied results in various levels of awareness about AI. The study has to be conducted on a large population of dentists to know the KAP of AI among dentists in Tamil Nadu.

## Conclusions

The present study shows that awareness of AI is not satisfactory. Therefore, awareness about AI has to be achieved through dental associations, research institutions, and technology companies by promoting discussions and educational resources related to AI. Research papers, publications, and presentations on AI in dentistry could contribute to spreading awareness among dental students. The field of AI is rapidly evolving, and new applications and tools are being developed regularly. Thus, staying updated on advancements in AI is essential for dentists interested in incorporating AI into their work. To improve the future of dentistry in this revolutionary AI period, the curriculum has to be updated.
